# Considerations for cell passaging in cell culture seed trains

**DOI:** 10.1186/1753-6561-9-S9-P43

**Published:** 2015-12-14

**Authors:** Tanja H Rodríguez, Ralf Pörtner, Björn Frahm

**Affiliations:** 1 Biotechnology & Bioprocess Engineering, Ostwestfalen-Lippe University of Applied Sciences, Lemgo, D-32657, Germany; 2 Institute of Bioprocess and Biosystems Engineering, Hamburg University of Technology, Hamburg, D-21073, Germany

## Background

The purpose of a seed train is the generation of an adequate number of cells for the inoculation of a production bioreactor. This is time- and cost-intensive. From volumes used for cell thawing or cell line maintenance the cell number has to be increased. The cells are usually run through many cultivation systems which become larger with each passage.

The seed train steps have a significant impact on the product titer and cell growth in production scale, as well as the success and the reproducibility of the seed train itself. Furthermore, cell line changes in the existing facility require adaptions of the seed train protocol.The design of a new facility involves the choice of the optimal seed train scales in order to meet the future requirements of the cultivated cell lines.

## Motivation

A software tool is being developed which is able to mathematically describe different seed trains based on the user's input of the corresponding seed train information. This allows analysis and optimization of existing seed trains as well as design of new seed trains for new cell lines and design of seed train scales for new facilities [1]. An important challenge is to identify at which points in time the cells should be passaged from one scale into the next.

## Results

### Tool structure

The program structure of the tool is suitable for different seed trains. Currently, there are two embedded models describing cell growth, cell death, uptake of substrates and production of metabolites via a first order system of ordinary differential equations and mono d-type kinetics. Seed train simulation ispossible for different cell lines via entering corresponding model parameters (determined based on cultivation data using the Nelder-Mead algorithm). Furthermore, the tool offers different cell passaging criteria for seed train optimization.

### Strategies for cell passaging

For the investigated criteria for cell passaging, the cells are transferred into the next scale according to the following four different methods.

Method A: Based on empirical experience respectively a standard operating procedure, a fixed time span is used for cell passaging, e.g. when cell growth in the first scale reached 80 % of the maximum cell concentration.

Method B: In order to achieve an optimal Space-Time-Yield (STY), the point in time of optimal STY, *t_STY,opt _*in each scale is used for cell passaging, with *STY *= (*Viable cell concentration*)/*t*.

Method C: This method uses the point in time when the apparent growth rate decreases to a before defined percentage (e.g. 90%): *t*_*μ*90%_:= *t*(*μ_app_*= 0.9·*μ_max_*) In this way a high apparent growth rate can be guaranteed.

Method D: This method combines two criteria - the point in time of optimal STY (method B) and the point in time when the effective growth rate decreases to a before defined percentage (method C). Then the average of both points in time is calculated and chosen as point in time for cell passaging: $\left(t_{STY_{opt}}+t_{\mu 90\%}\right)/2$. This procedure combines a high STY with a high effective growth rate.

### Application example

**Figure 1:**
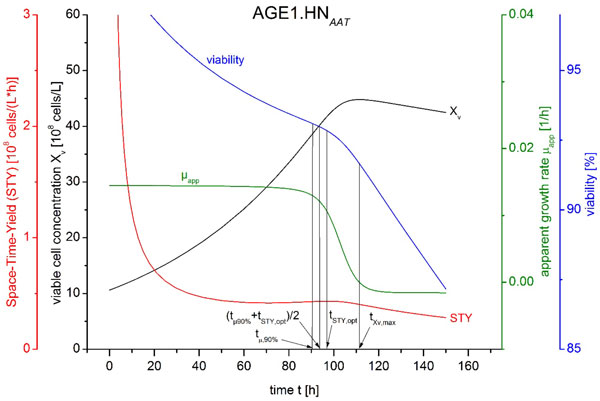
**Courses of viable cell concentration, Space-Time-Yield (STY), apparent growth rate and viability over cultivation time in flask scale 1 as an example as well as four different points in time - three points in time for cell passaging according to the methods B-D and the point in time of maximum viable cell concentration**.

Figure 1 illustrates the different cell passaging criteria for the cell line AGE1.HN_AAT_(ProBioGen AG). Presented are the simulated courses of viable cell concentration, Space-Time-Yield (STY), apparent growth rate and viability at the beginning of the seed train.

The point in time of optimal Space-Time-Yield (STY) corresponds to an apparent growth rate of 74 % of its maximum. In experimental lab scale seed train cultivations cell growth in the next scale was slowed down [2]. Therefore, method B (cell passaging at point in time of optimal STY) is not recommended. The point in time of apparent growth rate decreased to 90 % of its maximum (method C) corresponds to a viable cell concentration of 86 % of its maximum. The average of point in time of optimal STY and point in time of apparent growth rate decreased to 90 % (method D) corresponds to a viable cell concentration of 91 % of its maximum and an apparent growth rate of 83 % of its maximum.

## Conclusions

Seed trains which usually last in the range of weeks can be simulated within a short time. Different cell passaging criteria can be analysed such as the four examples described above. Transfer of AGE1.HNAAT cells at the maximum Space-Time-Yield into the next scale was not advantageous since at this point in time the apparent growth rate was decreased so far that growth in the next scale was already slowed down.

## Acknowledgements

The cell line AGE1.HN was kindly provided by ProBioGen AG, Berlin, Germany.
